# Selection of extended CRISPR RNAs with enhanced targeting and specificity

**DOI:** 10.1038/s42003-024-05776-8

**Published:** 2024-01-12

**Authors:** Ashley Herring-Nicholas, Hillary Dimig, Miranda R. Roesing, Eric A. Josephs

**Affiliations:** Department of Nanoscience, The University of North Carolina at Greensboro, Greensboro, NC USA

**Keywords:** Targeted gene repair, Genetic engineering, Synthetic biology

## Abstract

As CRISPR effectors like Cas9 increasingly enter clinical trials for therapeutic gene editing, a future for personalized medicine will require efficient methods to protect individuals from the potential of off-target mutations that may also occur at specific sequences in their genomes that are similar to the therapeutic target. A Cas9 enzyme’s ability to recognize their targets (and off-targets) are determined by the sequence of their RNA-cofactors (their guide RNAs or gRNAs). Here, we present a method to screen hundreds of thousands of gRNA variants with short, randomized 5’ nucleotide extensions near its DNA-targeting segment—a modification that can increase gene editing specificity by orders of magnitude—to identify extended gRNAs (x-gRNAs) that effectively block any activity at those off-target sites while still maintaining strong activity at their intended targets. X-gRNAs that have been selected for specific target / off-target pairs can significantly out-perform other methods that reduce Cas9 off-target activity overall, like using Cas9 variants engineered for higher specificity in general, and we demonstrate their effectiveness in clinically-relevant gRNAs. Our streamlined approach to efficiently identify highly specific and active x-gRNAs provides a way to move beyond a one-size-fits-all model of high-fidelity CRISPR for safer and more effective personalized gene therapies.

## Abstract (web summary)

CRISPR-Cas enzymes are increasingly entering clinical trials for gene therapies but can sometimes alter DNA off-target. We present an approach to modify the guide RNA to effectively eliminate off-target activity for target sequences of interest.

## Introduction

CRISPR effector Cas9 from *Streptococcus pyogenes* (SpyCas9) has emerged over the past several years as a powerful biotechnological tool that also holds tremendous therapeutic potential in the treatment of genetic diseases, including several that have entered clinical trials^1,2^. This potential arises from the ability of CRISPR effectors to use a modular segment of their RNA co-factor (their guide RNA or gRNA) to recognize DNA sequences complementary to its DNA-targeting segment (called the ‘spacer’) and introduce targeted mutations into the DNA at those sites^3,4^. However, oftentimes a gRNA for a specific target can cause the Cas9 nuclease to introduce off-target double-strand breaks (DSBs) and mutations at similar nucleotide sequences that are also present elsewhere in that genome^5,6^. The possibility of unintended Cas9-induced mutational events raises significant concerns for therapeutic applications. The challenge in preventing these off-target mutations lies in the fact that human genome is large, that Cas9 effectors are tolerant to small differences between the intended target and other sites that might be present in an individual’s genome^7^, and the existence of these off-target sites in a patient’s DNA may not even be known or detected a priori. In particular, it is becoming increasingly important to recognize that individuals carry unique or personal off-target sequences for a therapeutic gRNA as a result of genetic variations that exist between people and/or across different populations, and that these unique off-target sequences must be accounted for in an era of personalized medicine^8,9^.

There are a few ways to reduce Cas9’s off-target activity overall and increase the specificity of CRISPR systems in general. For example, these general approaches include reducing cellular exposure to Cas9 nucleases^10^ or selectively inhibiting Cas9 nuclease activity altogether^11^, as well as using engineered, high-fidelity (or enhanced-specificity) Cas9 variants such as eCas9^12^. eCas9 effectors have amino acid substitutions designed to reduce their overall affinity for DNA to decrease both the probability that the effectors’ latent nuclease domains will become activated at sequences with imperfect complementarity to its gRNA^13^ and their overall activity^14^. Modification of the gRNA itself has also been found to modulate Cas9 specificity; gRNAs with chemically modified bases, phosphates, or sugars can exhibit increased specificity overall relative to unmodified gRNAs, although the optimal combination of modifications for a specific target/off-target can be difficult to predict de novo^15,16^. Removing a few nucleotides from the 5’ end of the spacer of the gRNA (from 20 nt to 17–18 nt) to generate truncated gRNA (tru-gRNAs) can also decrease off-target activity^17^, an effect likely caused by a general destabilization of gRNA/DNA interactions when the spacers are shortened^13^. There have also been other approaches attempt to ‘mask’ potential off-targets with the co-transfection of additional CRISPR components and/or gRNAs^18,19^.

**Fig. 1:**
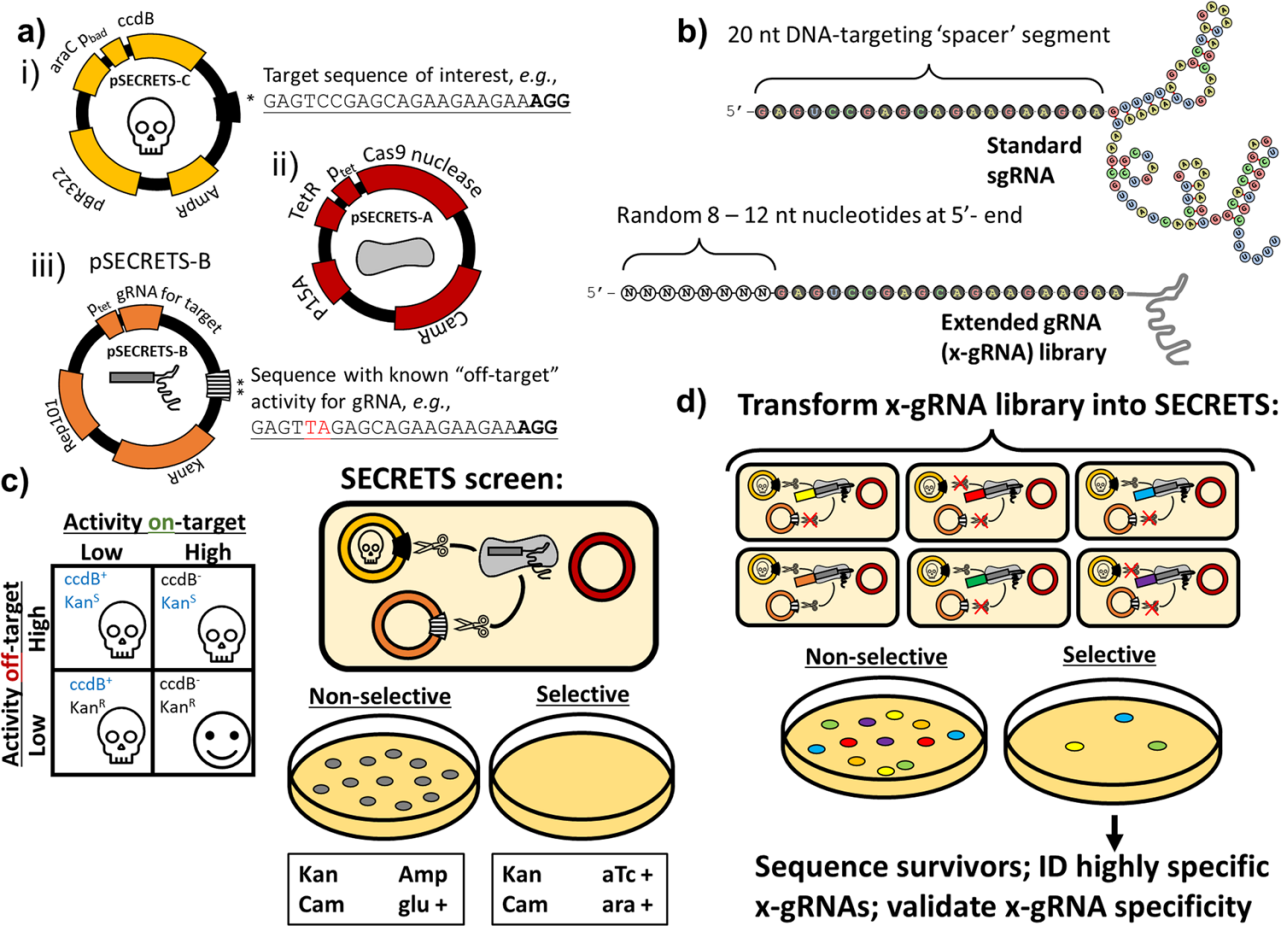
SECRETS screen to identify short 5’-nucleotide extensions to gRNAs that increase Cas9 gene editing specificity for a given target with known off-targets. **a** Simplified schematics of the three SECRETS plasmids: (i) high-copy number plasmid with inducible toxin and target sequence of interest to select for on-target activity, (ii) medium copy plasmid for inducible expression of Cas9, and (iii) low-copy plasmid for gRNA expression and counterselection for off-target activity with kanamycin resistance cassette and a known off-target sequence for the gRNA. **b** (above) a standard gRNA has 20 nt DNA-targeting segment known as a spacer while (below) an extended gRNA (x-gRNA) has an additional 8–12 nt to the 5’- of the spacer. **c** During SECRETS selection, *E. coli* expressing standard gRNAs that exhibit promiscuous activity are unable to survive. **d** Members of large, randomized x-gRNA libraries can be screened simultaneously using SECRETS for 5’-extensions that result in efficient on-target Cas9 activity and minimized off-target activity that can be used for highly specific gene editing applications at that target.

Recently, it was found that adding short nucleotide extensions (~6 to ~16 nts) to the 5’-end of the gRNA spacer (Fig. 1)—especially those that were predicted to form ‘hairpin’ or secondary structures with the spacer designed to interfere with gRNA interactions at specific off-target sequences—could significantly reduce Cas9 off-target activity while maintaining on-target mutational efficiencies^20^. On average, the specificity in targeting during gene editing for these gRNA variants termed hairpin-gRNAs (hp-gRNAs) increased 50-fold (and up to 200-fold) relative to gene editing using standard gRNAs, and this approach worked in diverse CRISPR effectors for multiple target sites each. While those hp-gRNAs could significantly outperform other existing engineered gRNAs, the 5’ extended sequences were each designed and tested one at a time, manually, for each targeted sequence and set of off-targets. At the time, it was also found that some of tested 5’-extensions did not effectively reduce off-target activity, others also significantly inhibited on-target activity, and still others that were not predicted to increase specificity (controls) occasionally did. Because there are a very large number of possible short 5’-extensions and in principle, different 5’-extensions for the same spacer sequence can be fine-tuned or optimized to limit activity versus specific off-target sequences, the inability to predict de novo which of those sequences will increase the specificity of an associated Cas9/gRNA ribonucleoprotein (RNP) has limited their utility in practice in eliminating the risk of off-target mutation during gene editing.

To overcome this challenge, here we present an experimental protocol to simultaneously screen tens- to hundreds- of thousands of candidate 5’-extension sequences to efficiently and reliably identify novel extended gRNA sequences (x-gRNAs) that maintain robust Cas9 activity on-target while significantly increasing gene editing specificity by effectively eliminating its activity at known off-target sequences where conventional approaches to increase general Cas9 specificity may fail (Fig. 1). In this protocol, called Selection of Extended CRISPR RNAs with Enhanced Targeting and Specificity (SECRETS), the activity of Cas9 enzymes with a library of x-gRNA candidates are evaluated in parallel using an *Escherichia coli*-based system that is strongly selective for the ability to stimulate Cas9 nuclease activity on-target and counter-selective for activity at their off-targets. We found that x-gRNAs generated using SECRETS for specific target/off-target pairs can significantly out-perform other methods that reduce Cas9 off-target activity overall, like using Cas9 variants that have been engineered for higher specificity in general for several high-activity and clinically relevant gRNAs. Our streamlined approach to efficiently identify highly specific and active x-gRNAs provides a way to move beyond a one-size-fits-all model of high-fidelity CRISPR for safer and more effective personalized gene therapies.

## Results and discussion

### The SECRETS protocol to efficiently identify high-activity and high-specificity extended gRNA (x-gRNA) variants

To perform SECRETS, an *E. coli* strain is prepared to propagate three plasmids (Fig. 1a): (i) a high-copy plasmid expresses the toxin ccdB in the presence of arabinose (ara), and also contains the target sequence of interest; (ii) a medium-copy plasmid expresses Cas9 in the presence of anhydrotetracycline (aTc) and provides chloramphenicol resistance; and (iii) a low-copy plasmid expresses the x-gRNAs library (Fig. 1b), provides resistance to the antibiotic kanamycin (KanR), and also contains a known off-target sequence for the gRNA. DSBs induced by the Cas9 at the target of interest in *E. coli* results in the degradation of ccdB plasmid, allowing the bacteria to survive in the presence of arabinose, while DSBs induced by Cas9 at the known off-target results in the degradation of the gRNA plasmid and bacterial susceptibility to the antibiotic kanamycin (KanS)^21^. We note that the imbalance in plasmid copy number^22^ is intentional: while the ‘high-copy’ plasmids with their targets must all be degraded in order to remove the toxin—which will require high levels of activity by the CRISPR RNP— there is less margin for error with the ‘low-copy’ off-target plasmid, such that RNPs with even a low rate of target cleavage might result in the total removal of that plasmid. This has the effect of making the process highly selective against any activity off-target, as fewer cleavage events might result in susceptibility to antibiotics. Hence, only gRNAs that exhibit robust activity at their intended targets and low activity at their off-target sites will survive a SECRETS screen (Fig. 1c)^23^.

### SECRETS identifies optimized x-gRNAs for diverse target/off-target pairs

**Fig. 2:**
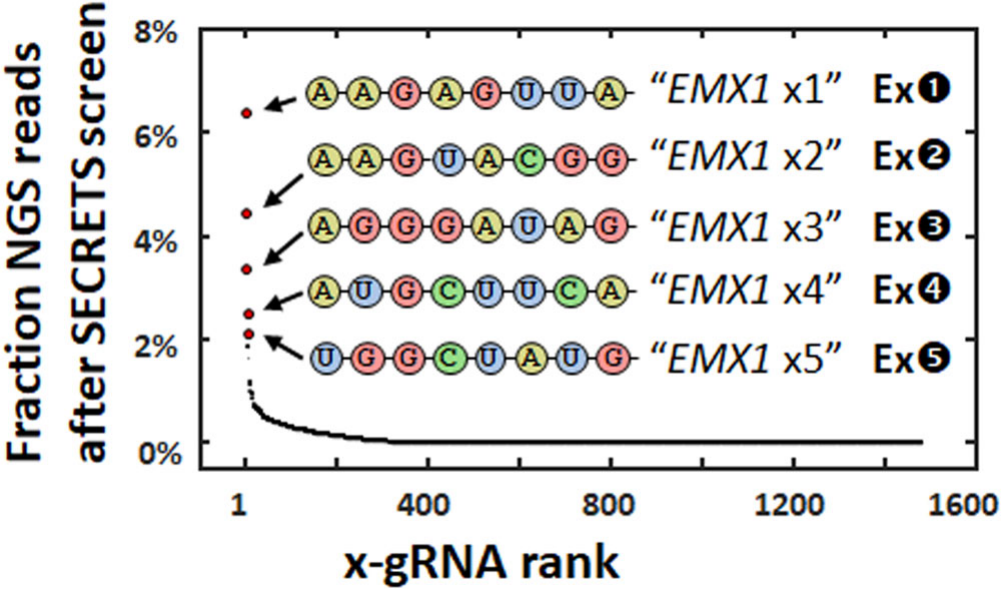
Next-generation sequencing following SECRETS screens reveals candidate x-gRNAs for *EMX1* with high activity and specificity. The average fraction of next-generation sequencing (NGS) reads from two SECRETS replicates reveals several standout x-gRNAs from *E. coli* that survived (Ex1-5). These top 5 (red dots) were subsequently evaluated for subsequent in vitro validation.

**Fig. 3:**
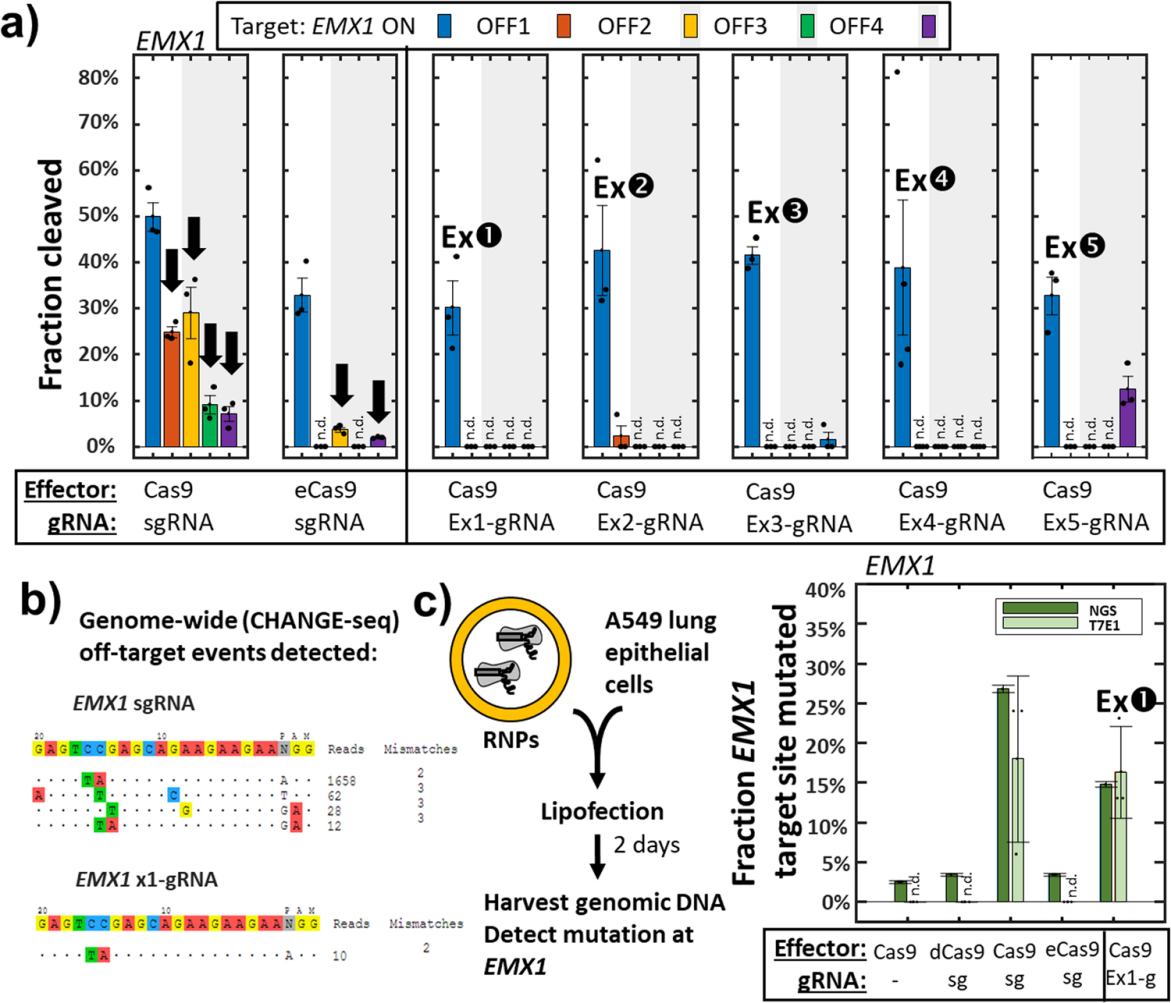
Validation of x-gRNAs identified from SECRETS. **a** All of the top five x-gRNAs exhibit remarkable on-target activity (comparable or greater than enhanced-specificity Cas9 variant eCas9) and greatly reduced off-target activities across four known off-target sites for the standard x-gRNA during in vitro cleavage assays using purified Cas9 and in vitro-transcribed (x-)gRNAs. *n* = 3 replicates each, dots are individual data points. Error bars = standard error of the mean. Black arrows highlight off-target activity of Cas9 and eCas9 with the sgRNA that was blocked when using x-gRNAs. **b** CHANGE-seq^5^ reveals that x-gRNAs identified through SECRETS exhibit minimal and greatly reduced genome-wide off-target activity, with no novel off-target activity identified relative to the standard gRNA. *n* = 2 biological replicates each. **c** x-gRNAs identified through SECRETS exhibit robust genome editing activity following transfection of purified Cas9 ribonucleoprotein (RNP) complexes. sg standard gRNA, dCas9 catalytically inactive Cas9, eCas9 enhanced specificity Cas9, NGS next-generation (illumina) sequencing, T7E1 T7E1 mutation detection assay. *n* = 3 biological replicates, 2 NGS replicates, and 3 T7E1 replicates. Error bars = standard error of the mean.

**Fig. 4:**
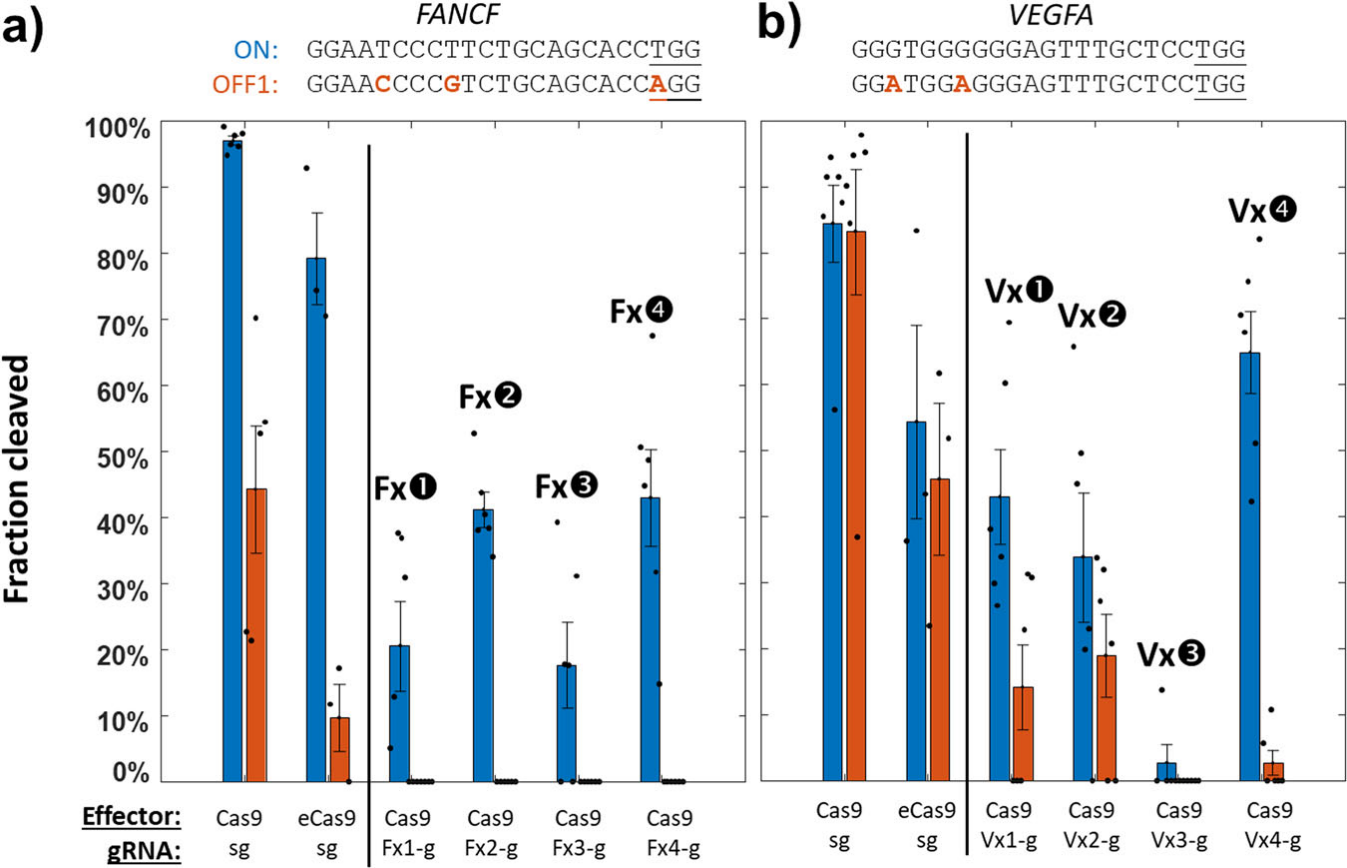
SECRETS can identify high-specificity x-gRNAs that outperform eCas9. **a** Highly specific x-gRNAs could also be readily identified from SECRETS screens for gRNAs for *FANCF* and **b** *VEGFA* targets. Note significant off-target activity even with eCas9 with the *VEGFA* gRNA that was blocked by the Vx4-gRNA, with comparable on-target activity, and FANCF x-gRNAs have no detectable off-target activity. *n* = (at least) 3 replicates each, dots are individual data points. Error bars = standard error of the mean. eCas9 = enhanced specificity Cas9. See the activities of the full set of the top 4 FANCF and VEGFA x-gRNAs in Fig. S3.

We tested this system with a well-characterized gRNA^20^ for human gene *EMX1* against its target sequence (*EMX1* ON) and another sequence within the human genome where the Cas9/gRNA RNP complex is known to exhibit off-target activity (*EMX1* OFF1) due to a two-nucleotide difference at positions where Cas9 effectors are especially susceptible to tolerating sequence divergence (Fig. 1a)^24^. After only 1 hr of Cas9/gRNA expression, followed by plating and overnight growth on LB with aTc, arabinose, chloramphenicol (cam), and kanamycin, we find strong suppression of *E. coli* growth with the standard *EMX1* gRNA, but only when the *EMX1* OFF1 sequence is present in the kanamycin resistance plasmid (Fig. S1). If instead of the standard gRNA for *EMX1* (Fig. 1b top) we introduce a library of *EMX1* x-gRNA variants with 8 randomized nucleotides (N8) appended to its 5’ end (Fig. 1b bottom), we find numerous *E. coli* colonies of survivors of the SECRETS protocol (Figs. 1d and S1), indicating that these x-gRNAs from the library demonstrate the high Cas9 activity and specificity required to survive. The pooled survivors were sequenced and each of the top five most prevalent x-gRNA sequences in the surviving population (Fig. 2) were tested for activity and specificity in vitro. In vitro Cas9 digestion assays revealed nuclease activities of Cas9 with all five of the x-gRNAs identified from the SECRETS screen were significantly reduced at *EMX1* OFF1 compared to the activity of the standard *EMX1* gRNA—effectively eliminating nuclease activity at the known off-target site—and exhibited similar activity at *EMX1* ON as the engineered (enhanced-specificity) Cas9 variant eCas9 (Figs. 3a and S2; Supplementary Data 2). These five x-gRNAs identified through the SECRETS protocol also exhibited higher levels of specificity in general, eliminating Cas9 off-target activity across three other known *EMX1* off-targets (*EMX1* OFF2 – OFF4, containing 2–4 differences with the *EMX1* ON sequence), and reducing nuclease activity at all four off-target sequences even more so relative to eCas9 with a standard gRNA (Figs. 3a and S2). In addition to x-gRNAs for *EMX1*, at which site the regular gRNA for EMX1 exhibits relatively low (~50%) activity in vitro, we also tested whether SECRETS could be used to efficiently identify multiple x-gRNAs for ‘high-activity’ targets—human genes *VEGFA* and *FANCF* (Figs. 4, S3, and Supplementary Data 2)^20^—with superior activity and specificity profiles (Fig. 4), including in the case of *VEGFA* where eCas9 was not able to significantly reduce activity at OFF1 sites.

To confirm that the x-gRNAs identified via SECRETS would remain active in human cell lines for genome editing, we transfected RNP complexes with Cas9 variants (wild-type Cas9; catalytically inactive dCas9^4^; or engineered enhanced-specificity eCas9^12^) and gRNA variants (standard gRNAs or x-gRNAs) into A549 human lung epithelial cells, then performed T7E1 mutation detection assays and next-generation sequencing (NGS) to quantify mutation rates at the *EMX1* target. The Cas9 RNPs with x-gRNAs identified *via* SECRETS exhibited robust genome editing in A549 cells, and higher on-target mutation rates than eCas9 (Fig. 3c). While it had been previously reported that extensions to gRNAs outside of the segment of the spacer used for DNA recognition and targeting did not introduce any novel off-targets^20^—likely because the 20 nt targeting segments of the gRNA remain the same—to ensure that no new off-targets were introduced when using x-gRNAs identified from the SECRETS screen we also performed a test of genome-wide off-target nuclease activity (CHANGE-seq^5^). As expected, we found significant reductions of off-target cleavage activity genome-wide and no new off-targets (Fig. 3b). Therefore, these findings demonstrate that the SECRETS protocol can robustly identify multiple high-performance x-gRNA candidates with strong potential for specific gene editing applications in human cells that eliminate off-target activity at selected loci.

### SECRETS identifies optimized x-gRNAs for therapeutically relevant targets

**Fig. 5:**
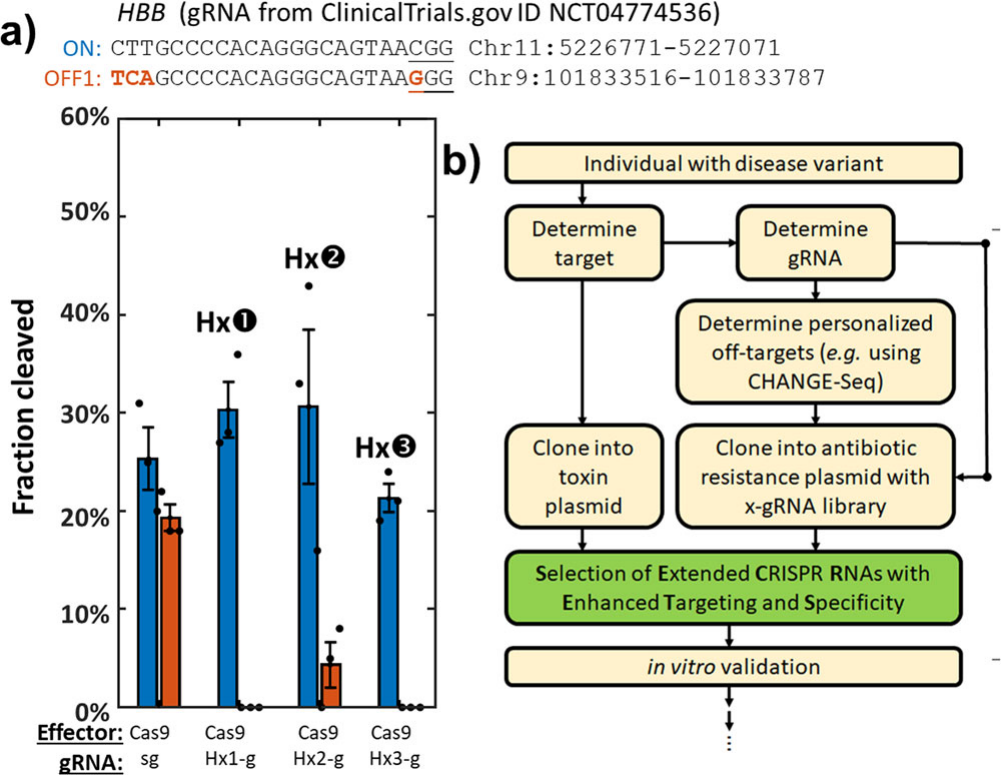
SECRETS is effective at identifying highly specific x-gRNAs for clinically relevant gRNAs. **a** In vitro validation of 3 x-gRNAs for a gRNA used in a treatment of sickle-cell disease (SCD) targeting the *HBB* gene from ClinicalTrials.gov ID NCT04774536 (see main text). While the regular gRNA exhibits significant off-target activity, x-gRNAs identified *via* SECRETS were able to completely abolish detectable off-target activity while maintaining activity on-target comparable to the standard gRNA. **b** A workflow for how SECRETS might improve the safety in the protocols for developing personalized gene therapies. Once an individual has been recognized as a candidate for gene therapy and a target for Cas9 determined, the target is used to determine a high-efficiency gRNA sequence to be used for therapy and itself cloned into the plasmid with a toxin (pSECRETS-C). The gRNA is then used to screen for sequences where off-target activity might occur specifically in that individual’s genome (e.g. using CHANGE-Seq^5^) and cloned into the x-gRNA library plasmid with a kanamycin resistance cassette (pSECRETS-B) along with the off-target sequence(s) to avoid. After SECRETS, sequencing, and in vitro validation, highly specific x-gRNAs for that personalized gene therapy will have been efficiently generated.

Given these findings, we next sought to determine whether the SECRETS protocol could be applied to gRNAs with potential therapeutic applications, such as one currently being used in a clinical trial for the treatment of sickle-cell disease (SCD) (Walters, M. (2024-06-01 (Est.) - 2028-06-01 (Est.)). Transplantation of Clustered Regularly Interspaced Short Palindromic Repeats Modified Hematopoietic Progenitor Stem Cells (CRISPR_SCD001) in Patients With Severe Sickle Cell Disease. Identifier NCT04774536. https://clinicaltrials.gov/study/NCT04774536). This particular gRNA^25,26^ targets the *HBB* gene to perform homology-directed correction of the SCD variant but has also been reported to exhibit significant levels of activity a known off-target, which we also observed in vitro. In this case, the off-target differs from the target by 3 nucleotides that would base-pair with the extreme 5’- end of the gRNA (Fig. 5), the position where Cas9 RNPs are most tolerant to mismatches and a position where other techniques like truncating the 5’- end of the gRNA^17^ would be likely to improve specificity. We performed a SECRETS screen then tested the top three x-gRNAs in vitro (Supplementary Data 3), where we found that in fact, they did dramatically reduce activity at the off-target sequence while maintaining the activity on-target of the standard gRNA (Fig. 5a). This demonstrates that the SECRETS protocol can be applied to therapeutically relevant gRNAs and potentially make them safer with significantly reduced risks of off-target mutations.

### Further development of the SECRETS protocol

In the demonstrations above, we screened randomized x-gRNA libraries of 65,336 variants (N8) in the SECRETS protocol for each gRNA target. We also identified enhanced x-gRNAs from more complex initial libraries (250,000 + 5’- extension sequence variants), including pooled libraries of N8 variants containing different additional 4 nt tetraloop motifs^20^ (N8+4) designed to promote interactions between the N8 segment and the DNA-targeting segment of the x-gRNA (Fig. S4). Indeed, we note that the ‘space’ of potential 5’-extension sequences for x-gRNAs is quite large^20^ (Fig. S4A)—we expect larger x-gRNA libraries of 4^10^ or 4^12^ (1–16 M) variants can be quite readily generated and screened in *E. coli* for new targets/off-target pairs of interest. The results here suggest that this space is also quite rich with high performance x-gRNA variants, as we could identify multiple x-gRNAs for several spacer sequences under relatively small-scale SECRETS screens that exhibited exceptional activity and specificity profiles. Furthermore, hp-gRNAs were able to improve the specificity of diverse CRISPR effectors, including Cas9 from *Staphylococcus aureus* and various Cas12 effectors^20^, the SECRETS protocol can be readily adapted to those systems as well (Fig. S4A).

## Conclusions

For many biotechnological or therapeutic applications, it is often desirable to direct a Cas9/gRNA RNP to a specific nucleotide target of interest (e.g., where there might be no flexibility to target nearby sites). If it is determined that there is a potential for off-target activity at certain sites in a sample or for a patient, there can be limited options to minimize activity at those specific off-targets. Here we demonstrate that the SECRETS protocol can be used to robustly identify ultra-specific variants for those gRNAs of interest and clinically relevant gRNAs that have been explicitly counter-selected against activity at those off-target sites. This approach could be used to robustly generate x-gRNAs in a *design-free* way that effectively eliminates the need for individualized optimization and has been experimentally streamlined for simplicity by cloning new target/off-target pairs on-demand into the screening plasmids for ease of rapidly selecting enhanced x-gRNAs. Once any potential off-target sites have been determined, the SECRETS screen therefore provides an accessible and reliable method to identify high-performance x-gRNA variants for specific targets of interest (Fig. 5b). We expect the continued output and development of this approach will allow for safer applications of advanced CRISPR gene editing approaches that require gRNAs with extreme specificity, such as SNP-targeting and/or allele-specific gene editing. As approaches for predicting and identifying novel off-target sequences in individual patients become more sophisticated and routine^5,8^, we expect methods like SECRETS, which can reliably and rapidly generate highly specific and highly active gRNA variants that effectively eliminate Cas9 activity at specific off-targets, will become increasing important in applications of gene therapy and personalized medicine.

## Methods

### DNA oligonucleotides, dsDNA, and plasmids

DNA sequences for all oligonucleotides, dsDNA fragments, and plasmids are listed in Supplementary Data 4, Supplementary Data 5, and Supplementary Note 1, respectively.

### Cell lines and *E. coli* strains

All cloning was performed using New England Biolabs (NEB) 10-beta cells (NEB #C3020K) or TOP10 (Invitrogen #C404010) cells, and all SECRETS assays performed in Stbl2 cells (Invitrogen #10268019), grown at 30 °C. A549 (ATCC CCL-185™) human lung epithelial cells were obtained from ATCC (American Type Culture Collection).

### Cloning SECRETS plasmids and x-gRNA libraries

#### SECRETS plasmids

Three plasmids were generated for the validation of the SECRETS protocol: pSECRETS-A (medium copy p15A ori, chloramphenicol resistance, aTc-inducible Cas9 expression; Fig. 1aii; Addgene Plasmid #196986), pSECRETS-B (low copy SC101 ori, kanamycin resistance, aTc-inducible gRNA expression, and a site for off-target sequence; Figure 1Aiii; Addgene Plasmid #196987), and pSECRETS-C (p11.LacY.wtx1 plasmid (Addgene #69056) high copy pBR322 ori, ampicillin resistance, arabinose-inducible/glucose-suppressed ccdB toxin also containing additional site for the target sequence; Fig. 1ai). p11-LacY-wtx1 was a gift from Huimin Zhao (Addgene plasmid # 69056; http://n2t.net/addgene:69056; RRID:Addgene_69056).

To clone pSECRETS-A, the Cas9 gene was PCR amplified from pwtCas9-bacteria (Addgene #44250) and a gBlock (purchased from Integrated DNA Technologies [IDT]) containing an anhydrotetracycline (aTc) inducible promoter (pLTetO-1) was inserted via HiFi Assembly (HiFi Assembly Kit (NEB#E5520S)) into a PCR-amplified fragment of plasmid pBbA2c-RFP (Addgene #35326) to replace the red fluorescent protein (RFP) gene. pwtCas9-bacteria was a gift from Stanley Qi (Addgene plasmid # 44250; http://n2t.net/addgene:44250; RRID:Addgene_44250). pBbA2c-RFP was a gift from Jay Keasling (Addgene plasmid # 35326; http://n2t.net/addgene:35326 ; RRID:Addgene_35326).

To clone pSECRETS-B, the gRNA cassette for aTc-induced expression was constructed by inserting *via* HiFi Assembly a PCR’ed fragment of TetR from pBbA2c-RFP (Addgene #35326) and a gBlock (IDT) containing the pLTetO-1 promoter and a Golden Gate cassette (dual BsaI restriction sites) near a Cas9 fused tracrRNA-crRNA fusion to replace the RFP and LacI genes in a PCR’ed fragment of pBbS2K-RFP (Addgene #35330). The Golden Gate assembly cassettes of the resulting plasmid (called pSECRETS-B) could then be used to clone spacer sequences or x-gRNA libraries for gRNA expression after BsaI digestion and ligation of short phosphorylated annealed oligos or HiFi Assembly of single-stranded oligos, respectively. pBbS2k-RFP was a gift from Jay Keasling (Addgene plasmid # 35330; http://n2t.net/addgene:35330; RRID:Addgene_35330).

To clone pSECRETS-B derivatives containing off-target sequences and standard gRNAs or x-gRNA libraries), single-stranded oligonucleotides (oPools) were synthesized by IDT containing the off-target sequences, pLTetO-1 promoter, (N8 random nucleotides immediately upstream) the spacer sequence. These oligos were of the form:


5'-CCACTGCTTACTGGCTTATCGGAAGGGATCGTCCTGACCCCG[Off-target sequence, 20 nt + 3 nt PAM]CCCCCTCCGTGGAGAAAATTTCCCTATCAGTGATAGAGATTGACATCCCTATCAGTGATAGAGATACTGAGCAC[5'- extension library, for example: NNNNNNNN][20 nt spacer sequence]GTTTTAGAGCTAGAAATAGCAAG-3'.


These inserts were PCR’ed with primers

SECRETS-FwdUSER 5'-GCAAG\deoxyU\TAAAATAAGGCTAGTCCG-3' and

SECRETS-RevUSER 5'-CTTGC\deoxyU\ATTTCTAGCTCTAAAAC-3'where deoxyU is a deoxyuracil modified, and the plasmid pSECRETS-B PCR’ed with primers:

Bv3-FwdUSER 5'-GCAAG\deoxyU\TAAAATAAGGCTAGTCCG-3' and

B-RevUSER 5'-GTGGG\deoxyU\TCTCTAGTTAGCCAGAG-3'

These inserts were then cloned into the pSECRETS-B cassette *via* USER cloning (NEB #M5505S). To maintain library diversity, after transformation, *E. coli* was recovered in 1 mL SOC media for 1 h without selection, then 0.5 mL of the media was reinoculated into 7 mL LB with kanamycin (50 μg/mL) and grown overnight. 5 mL of the transformants were then centrifuged, then miniprepped. pSECRETS-B plasmids were sequenced using variants of primers:

SECRETS-BSeq5 5'- [NGS adapter][barcode]-GAGCGGATACATATTTGAATG-3'

SECRETS-BSeq3 5'- [NGS adapter][barcode]- AAGTTGATAACGGACTAGCC-3'

The pSECRETS-C plasmids containing desired on-target sequences were constructed *via* HiFi assembly into the p11.LacY.wtx1 plasmid (Addgene #69056), which was double digested with XbaI and SphI, with a dsDNA fragment containing a target site (20 bp + PAM), 15 bp genomic context sequences flanking the target, and overhang sequences matching the digested plasmid. These fragments were constructed by PCRing short oligos with the form:


5'- ATAACAGGGTAATATCACGC[15 bp upstream genomic sequence context] +[20 bp target sequence + 3 bp PAM] +[15 bp downstream genomic sequence context]AAGCTTGGCTGTTTTGGCGG -3'


*E. coli* strains containing pSECRETS-C plasmids were grown in solutions supplemented with glucose (glu) to suppress leakage of arabinose-induced promoter until selection.

### The SECRETS protocol and analysis

#### Validating selection strength using standard gRNAs

For validation, pSECRETS-C itself or pSECRETS-C containing the *EMX1* target site and flanking sequences (pSECRETS-C-EMX1); pSECRETS-A; and pSECRETS-B to express the standard *EMX1* gRNA (pSECRETS-B-EMX1-gRNA) or pSECRETS-B-EMX1-gRNA containing *EMX1* OT1 were electroporated sequentially into electrocompetent NEB10beta *E. coli* cells and recovered in SOC media. For the last transformation with pSECRETS-B, recovery media was supplemented with 10 ng/mL aTc for pre-induction of sgRNA and Cas9. Following recovery for 1 h, cells were plated on LB agar plates under selective (aTc, arabinose, chloramphenicol, kanamycin) and non-selective (glucose, chloramphenicol, kanamycin, ampicillin) conditions and incubated for 24 h.

#### Selection of extended g-RNAs (SECRETS protocol)

pSECRETS-B plasmids containing x-gRNA libraries and the off-target site were screened similarly to the validation experiments with few changes. *E. coli* cells were transformed in two steps instead of three: *E. coli* strains containing pSECRETS-A plasmids were electroporated with corresponding B and C plasmid simultaneously (75 ng each DNA). Following recovery, cells were centrifuged at 4 °C and supernatant was replaced with fresh LB before inoculating 0.5 mL of the culture into 7 mL liquid LB for selective or non-selective conditions and grown overnight. After miniprep (NEB #T1010L) of the resulting cultures, samples were PCR’ed across the gRNA segment and prepared for Illumina next-generation sequencing using variants of primers SECRETS-BSeq5 and SECRETS-BSeq3.

#### Analysis of SECRETS outcomes

Small-scale next-generation sequencing (Amplicon-EZ; Azenta Inc.; at least 50,000 reads) of samples from the SECRETS assay was carried out. Custom code was written in MATLAB (Mathworks; Natick, MA) to extract and count the 5’-extensions from the x-gRNA sequence of each read; however, in principle, a short line of code can be written to the same effect following the approach found in Reference^27^. The number of unique 5’-extensions were enumerated per sample and normalized to the total number of reads per sample and averaged across technical replicates (*n* = 2). The normalized number of reads per 5’-extension were then averaged across biological replicates (*n* = 2), sorted from most prevalent to least, and the top five most prevalent 5’-extensions per gRNA selected for further characterization.

### In vitro validation of x-gRNAs

#### Cas9 ribonucleoprotein (RNP) generation

DNA oligos of sgRNAs and x-gRNAs were designed according to the EnGen sgRNA Synthesis Kit (NEB #E3322) to add 5’- T7 RNA polymerase promoter sequence and 3’- Cas9 crRNA sequence and were purchased from Integrated DNA Technologies IDT then resuspended to a stock concentration of 100 μM. If the (x-)gRNA did not have an initial 5’- dG necessary for T7 RNA polymerase transcription, one was added in the DNA oligo sequence. For sgRNA synthesis, oligos were diluted 100x (1 μM) then used with the EnGen sgRNA Synthesis Kit per manufacturer’s instructions. Cas9 RNPs were formed following the IDT Alt-R CRISPR-Cas9 System – In vitro cleavage of target DNA with ribonucleoprotein complex protocol (Option 2). Cas9 enzyme (Sigma Aldrich, #CAS9PROT-250UG), eCas9 enzyme (Sigma Aldrich #ESPCAS9PRO-50UG), or dCas9 enzyme (IDT Alt-R® S.p. dCas9 Protein V3 #1081066) and sgRNA were combined in equimolar amounts in Phosphate buffered saline, pH 7.4 - PBS (ThermoFisher, #10010023) and incubated at room temperature for 10 min. Following incubation, RNPs were stored at −80 °C or immediately used for in vitro digestion reactions.

#### In vitro digestion reactions

Three hundred (300) bp DNA targets containing the target sequence ~200 bp from one end and the flanking genomic context were synthesized by Twist Bioscience, PCR amplified using the provided universal primers, purified, and resuspended in nuclease-free water to 100 nM. Three technical replications of reactions were assembled in the following order: 7 μL nuclease-free water, 1 μL target DNA substrate (100 nM), 1 μL 10x Cas9 Nuclease Reaction buffer (200 mM HEPES, 1 M NaCl, 50 mM MgCl2, 1 mM EDTA (pH 6.5 at 25 °C)), 1 μL Cas9-RNP (1 mM), then incubated for 1 hour at 37 °C followed by proteinase K digestion (1 μL - 56 °C for 10 min; ThermoFisher, #EO0491). Products were resolved on a 3% agarose gel stained with SYBR Gold and analyzed using ImageJ.

### Evaluation of gene activity of transfected Cas9/x-gRNAs into human cell lines

Cells were transfected using the Lipofectamine CRISPRMAX Cas9 Transfection Reagent (ThermoFisher #CMAX00003) kit. Prior to transfection, A549 lung epithelial cells were plated in 24-well plates at 25% confluency in Dulbecco’s Modified Eagle’s Medium - DMEM (ATCC #30-2002) + 10% Fetal Bovine Serum - FBS (ATCC 30-2020) + 1% Penicillin-Streptomycin solution (ATCC 30-2300) and incubated for 24 h at 37 °C + 5% CO_2_. Following incubation, the media was removed and cells were washed with 1× PBS and replaced with fresh DMEM + 10% FBS. Cas9 RNP complexes were formed in a 1:1.2 molar ratio of Cas9 protein to sgRNA with Cas9 Plus reagent to a total volume of 25 μL in Opti-MEM Reduced Serum Medium (ThermoFisher #31985070) per reaction (*n* = 3). RNPs were added to a mix of 25 μL Opti-MEM I and 1.5 μL CRISPRMAX reagent per reaction, and following a 10 minute room temperature incubation, 50 μL was added to each well. Cells were then incubated at 37 °C + 5% CO_2_ for 48 h.

### Analysis of gene editing outcomes

Cells were processed as follows using the GeneArt Genomic Cleavage Detection Kit (ThermoFisher #A24372). Cell media was collected in a 1.5 mL Eppendorf tube. Remaining attached cells were washed with PBS then detached using TrypLe Express (ATCC 30-2300) trypsin and transferred to the corresponding Eppendorf tube for centrifugation at 1200 × *g* at 4 °C to pellet cells. Once the supernatant was discarded, pellets were resuspended in cell lysis buffer with protein degrader (supplied in kit) and incubated at 68 °C then 95 °C to lyse cells. Crude cell lysate was mixed with forward and reverse primer (10 μM), AmpliTaq Gold 360 master mix, and nuclease-free water for direct PCR amplification of the region of interest followed by agarose gel electrophoresis to confirm expected PCR length. Heteroduplexes of the PCR products were formed by mixing with 10x detection buffer and heating samples to 95 °C, cooling to 85 °C at 2 °C/s, then to 25 °C at 0.1 °C/s. Detection enzyme was added to the samples and incubated at 37 °C, then fragments were resolved on a 3% agarose gel stained with SYBR Gold. Fluorescence was measured through ImageJ, intensity normalized by length of the DNA fragments, and fraction cleaved was determined using the following equation:

$$	[{{{{{\rm{sum}}}}}}\,{{{{{\rm{of}}}}}}\,{{{{{\rm{cleaved}}}}}}\,{{{{{\rm{band}}}}}}\,{{{{{\rm{intensities}}}}}}/\\ 	({{{{{\rm{sum}}}}}}\,{{{{{\rm{of}}}}}}\,{{{{{\rm{cleaved}}}}}}\,{{{{{\rm{and}}}}}}\,{{{{{\rm{parental}}}}}}\,{{{{{\rm{band}}}}}}\,{{{{{\rm{intensities}}}}}})]\times 100 \%$$

Two technical replicates of samples were also prepared for illumina next-generation sequencing and amplicon sequencing with editing efficiency determined using the CRISPResso2 pipeline^28^.

### Genome-wide off-target screens

We measured genome-wide off-target editing using CHANGE-seq described by Lazzarotto et al.^5^ with minor modifications. The genomic DNA (human male/female mixed, Promega #G3041) purification steps were carried out using the NEB Monarch Genomic DNA Purification Kit. An agarose gel was used to visualize the tagmentation of the human genomic DNA with the transposase. For the PCR step after cleavage and USER enzyme treatment (step 25 of the supplemental information), NEB 2X Q5 Master Mix was used in place of 2X Kapa HiFi HotStart Ready Mix. In place of the MiSeq protocol described in the supplemental information, cleaved genomic DNA barcoded and amplified via PCR for illumina sequencing was sent for NGS Amplicon-EZ sequencing by Azenta.

### Statistics and reproducibility

Technical and biological replicates as well as sample sizes are listed in their respective figure captions, typically two to three biological and two technical replicates. Statistical analysis such as two-sided *T*-tests and generation of confidence intervals were performed using MATLAB or Microsoft Excel.

### Reporting summary

Further information on research design is available in the Nature Portfolio Reporting Summary linked to this article.

### Supplementary information

Supplemental Materials

Description of Additional Supplementary Files

Supplementary Data 1

Supplementary Data 2-5

Reporting Summary

## Data Availability

Source data can be found for all graphs/charts presented in the main figures is found in Supplementary Data 1. Sequencing data is available at the Sequence Read Archive (SRA) with BioProject PRJNA1052720 with BioSamples SAMN38843455 : *EMX1* x-gRNAs; SAMN38843456 *FANCF* x-gRNAs; SAMN38843457 *VEGFA* x-gRNAs; SAMN38843458 : *HBB* x-gRNAs. All other data are available from the corresponding author on reasonable request.

## Materials availability

pSECRETS-A and pSECRETS-B precursors (without gRNA spacers or off-targets) are available from Addgene as Plasmid #196986 and Plasmid #196987, respectively. pSECRETS-C precursor plasmids (without on-target sequences) are available from Addgene (Addgene plasmid # 69056; http://n2t.net/addgene:69056; RRID:Addgene_69056) as a gift from Huimin Zhao.

## Code availability

Short MATLAB scripts used to extract 5’-extension sequences from NGS data will be provided upon request to the corresponding author.
