# Supplementary material for: Selection of extended CRISPR RNAs with enhanced targeting and specificity
Source: Commun Biol. 2024 Jan 12;7:86. doi: 10.1038/s42003-024-05776-8 (PMC10784525; doi:10.1038/s42003-024-05776-8)
Supplement: Supplementary file 2 — Description of Additional Supplementary Files [file 42003_2024_5776_MOESM2_ESM.pdf]

## Description of Additional Supplementary Files

**File name:** Supplementary Data 1
**Description:** The source data behind the graphs in the paper**.**

**File name:** Supplementary Data 2
**Description:** extensions from SECRETS protocol for EMX1, FANCF, and VEGFA Targets and top off-target. (Top five extensions sequences labeled as in Figures 3, 4, and S3).

**File name:** Supplementary Data 3
**Description:** extensions from SECRETS protocol for HBB target and top off-target from gRNA form clinical trial ClinicalTrials.gov ID NCT04774536. (top three extensions sequences labeled as in Figure 5A).

**File name:** Supplementary Data 4
**Description:** Oligonucleotide and primer sequences.

**File name:** Supplementary Data 5
**Description:** dsDNA fragments used to clone pSECRETS plasmids.
